# Multi-walled carbon nanotubes reversing the bone formation of bone marrow stromal cells by activating M2 macrophage polarization

**DOI:** 10.1093/rb/rbad042

**Published:** 2023-04-22

**Authors:** Runlian Lin, Kun Ge, Dehui Fan, Jing Li, Guoqiang Zhou, Kaihan Zhang, Yuanyu Huang, Lili Ma, Jinchao Zhang

**Affiliations:** Key Laboratory of Medicinal Chemistry and Molecular Diagnosis of the Ministry of Education, Key Laboratory of Chemical Biology of Hebei Province, College of Chemistry and Material Science, Hebei University, Baoding 071002, China; Key Laboratory of Medicinal Chemistry and Molecular Diagnosis of the Ministry of Education, Key Laboratory of Chemical Biology of Hebei Province, College of Chemistry and Material Science, Hebei University, Baoding 071002, China; Key Laboratory of Medicinal Chemistry and Molecular Diagnosis of the Ministry of Education, Key Laboratory of Chemical Biology of Hebei Province, College of Chemistry and Material Science, Hebei University, Baoding 071002, China; Key Laboratory of Medicinal Chemistry and Molecular Diagnosis of the Ministry of Education, Key Laboratory of Chemical Biology of Hebei Province, College of Chemistry and Material Science, Hebei University, Baoding 071002, China; Key Laboratory of Medicinal Chemistry and Molecular Diagnosis of the Ministry of Education, Key Laboratory of Chemical Biology of Hebei Province, College of Chemistry and Material Science, Hebei University, Baoding 071002, China; College of Basic Medical Science, Hebei University, Baoding 071000, China; Department of Chemistry, The University of Manchester, Manchester M13 9PL, UK; School of Life Science, School of Medical Technology, Advanced Research Institute of Multidisciplinary Science, Key Laboratory of Molecular Medicine and Biotherapy, Key Laboratory of Medical Molecule Science and Pharmaceutics Engineering, Beijing Institute of Technology, Beijing 100081, China; Key Laboratory of Medicinal Chemistry and Molecular Diagnosis of the Ministry of Education, Key Laboratory of Chemical Biology of Hebei Province, College of Chemistry and Material Science, Hebei University, Baoding 071002, China; Key Laboratory of Medicinal Chemistry and Molecular Diagnosis of the Ministry of Education, Key Laboratory of Chemical Biology of Hebei Province, College of Chemistry and Material Science, Hebei University, Baoding 071002, China

**Keywords:** multi-walled carbon nanotubes, bone microenvironments, osteogenesis, bone marrow stromal cells, M2 macrophages, cellular interactions

## Abstract

Multi-walled carbon nanotubes (MWCNTs) are an excellent bone tissue repair material both *in vitro* and *in vivo*. The interactions between MWCNTs and single type of cells of bone tissue, including osteoblasts, bone marrow stromal cells (BMSCs) or osteoclasts, have been extensively studied. However, the interactions between MWCNTs with different types of cells in the bone microenvironment remain elusive. Bone microenvironment is a complex system composed of different types of cells, which have interactions between each other. In this work, the effects of MWCNTs on bone microenvironment were firstly studied by culture of MWCNTs with BMSCs, osteoblasts, osteoclasts, macrophages and vascular endothelial cells, respectively. Then, co-culture systems of macrophages–BMSCs, macrophages–calvaria and macrophages–BMSCs–vascular endothelial cells were treated with MWCNTs, respectively. The osteogenic differentiation of BMSCs and osteoblasts was inhibited when these two types of cells were cultured with MWCNTs, respectively. Strikingly, when co-culture MWCNTs with BMSCs and macrophages, the osteogenesis of BMSCs was promoted by inducing the M2 polymerization of macrophages. Meanwhile, MWCNTs promoted the bone formation in the osteolysis model of calvaria *ex vivo*. In addition, the formation of osteoclasts was inhibited, and angiogenesis was increased when treated with MWCNTs. This study revealed the inconsistent effects of MWCNTs on single type of bone cells and on the bone microenvironment. The results provided basic research data for the application of MWCNTs in bone tissue repair.

## Graphical Abstract

**Figure:**
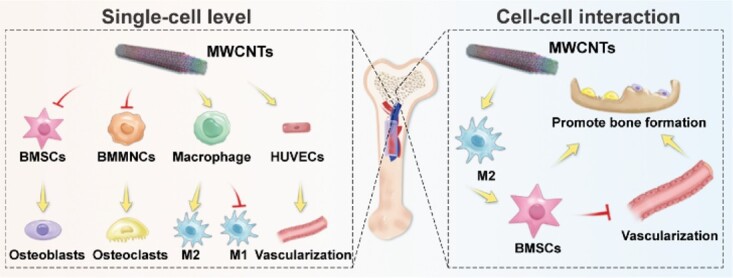

## Introduction

Multi-walled carbon nanotubes (MWCNTs) are an excellent bone tissue repair material, which has high biocompatibility and good mechanical properties. MWCNTs were reported to promote bone tissue repair both *in vitro* and *in vivo* [1–3]. However, these studies mainly focused on the interaction of MWCNTs and single type of cells in bone tissue, such as osteoblasts, bone marrow stromal cells (BMSCs) or osteoclasts [4–6]. Considering bone tissue is a complex system that contains multiple types of cells, there may be crosstalk between these cells. Therefore, it is important to reveal the network of MWCNTs and different types of bone cells.

Cells in the bone microenvironment include pericytes, osteoblasts, endothelial cells and others [7]. Pericytes are the most abundant cells in the bone microenvironment, accounting for about 67%, including BMSCs, bone marrow mononuclear cells (BMMNCs) and macrophages. Next are osteoblasts and endothelial cells, which account for 18% and 15%, respectively. BMSCs and osteoblasts play roles in bone formation. BMMNCs are recognized as the precursor cells of osteoclasts, which play a role in dissolving bone. Bone macrophages have two phenotypes, of which M1 type macrophages play a role in tissue defect and infection, and M2 type macrophages play a role in tissue regeneration. Endothelial cells construct the blood vessels, which nourish bone by transporting nutrients and oxygen. In bone microenvironment, bone formation is regulated by BMSCs, osteoblast and their interaction with other types of cells. Osteoblasts and osteoclasts regulate bone formation through the osteoprotegerin (OPG)/RANKL/RANK pathway. Nuclear factor-κB ligand (RANKL) and OPG are released from osteoblasts, and selectively bind to bone marrow monocyte membrane receptor RANK to determine whether the osteoclasts differentiation occurs [8]. Bone macrophages are close to the bone surface, and M2-type macrophages can secrete interleukin-4 (IL-4) to recruit BMSCs to promote bone formation by regulating the differentiation of BMSCs and osteoblasts [9–11]. In the complex bone microenvironment, the mechanisms of actions of MWCNTs on bone formation, especially the interplay between different types of cells, is still unknown. Therefore, it is necessary to take different types of bone cells and the bone microenvironment as a whole to study the effect of MWCNTs on bone tissue.

In this work, we tried to reveal the effect of MWCNTs on bone formation in the bone microenvironment. The carboxylated MWCNTs with a diameter of 20 nm and a length of 20–30 μm were used in the study. Firstly, BMSCs, osteoblasts (MC3T3-E1), BMMNCs, macrophages (Raw264.7) and human umbilical vein endothelial cells (HUVECs) were tested as selected cell models in the bone microenvironment to evaluate the effects of MWCNTs on cell viability, osteogenic differentiation and mineralization, osteoclast differentiation, macrophage phenotypic differentiation and vascularization. Next, BMSCs, macrophages and HUVECs were co-cultured to study the effect of BMSCs on the bone differentiation in bone microenvironment. Then, the calvaria of suckling mice co-cultured with macrophages under lipopolysaccharide (LPS) induction was established to study the regulation of bone microenvironment on bone formation at the sub-tissue level. The results provided basic research data for the application of MWCNTs in bone tissue repair.

## Materials and methods

### Materials and animals

Carboxylated MWCNTs were purchased from Chengdu Organic Chemistry Company, Chinese Academy of Sciences. CCK8 kit was obtained from Beyotime Biotechnology Company. Alkaline phosphatase (ALP) assay kit was purchased from Nanjing Jiancheng Institute of Biological Engineering. BCIP/NBT ALP color development kit was obtained from Beyotime. Alizarin Red, cetylpyridinium chloride and mouse recombinant macrophage colony-stimulating factor (M-CSF) were provided by Sigma-Aldrich. HiScript II Q RT SuperMix for qPCR (+gDNA wiper) and ChamQ Universal SYBR qPCR Master Mix were provided by Vazyme. ActinGreen 488 ReadyProbes, DAPI, arginase 1 (Arg-1) antibody, Dulbecco’s modified Eagle’s medium (DMEM), ɑ-MEM medium and high sugar DMEM (HDMEM) were ordered from Thermo Fisher Scientific. IL-4 and IL-6 enzyme-linked immunoassay (ELISA) kits were purchased from Shanghai Enzyme-Link Biotechnology. Inducible nitric oxide synthase (iNOS) antibody was provided from Abcam. Matrigel was purchased from BD Biosciences. Mouse recombinant RANKL was purchased from R&D Systems. LPS and penicillin/streptomycin were ordered from Solarbio. Tartrate-resistant acid phosphatase (TRAP) detection kit was provided by Wako. Osteo Assay Surface plates and transwell plates were purchased from Corning.

Four-week and pregnant ICR mice were purchased from Beijing Vital River Laboratory Animal Technology (Beijing, China). All the animal experiments were carried out at the Comprehensive Medical Experimental Center of Hebei University according to the Animal Welfare and Ethical Committee of Hebei University (Approval No. IACUC-2018025).

### Cell culture and cell viability evaluation

BMSCs and osteoblasts cell line MC3T3-E1 were used as the osteogenic cells. BMMNCs were used for the study of osteoclast differentiation and formation. Macrophages cells Raw264.7 were used to evaluate the phenotypic differentiation of bone macrophages. Furthermore, HUVECs were used as the model to test angiogenesis. The detailed culture methods of these cells were listed in the Supplementary Material.

CCK8 and MTT assays were employed for cell viability test [12, 13]. For the detailed methods, refer to the Supplementary Material.

### Osteogenic differentiation of BMSCs and MC3T3-E1

ALP and mineralization are important markers in osteogenic differentiation. According to the previous work, the staining and quantitative analysis of ALP and mineralization nudes were detected [14]. Actin was visualized by action green staining to reveal cytoskeleton change occurring in osteogenic differentiation. Cytoskeleton change also occurs in osteogenic differentiation. The actin was visualized by actin green staining [15] and the mobility of cells was detected by the transwell assay [16]. Osteogenesis-related genes were detected by qPCR and the primers are shown in Supplementary Table S1. Moreover, the detailed methods were all included in the Supplementary Material.

### Osteoclast differentiation of BMMNCs

The purified BMMNCs were seeded in a 48-well plate with the density of 5 × 10^5^ cells/well. After osteoclasts induction with M-CSF and RANKL, BMMNCs were treated with MWCNTs (0, 1 and 5 μg/ml) for 12 days, and the cells were washed with PBS, then fixed in 4% paraformaldehyde at 4°C for 10 min and stained with TRAP under the instruction of kit. The TRAP-positive cells were observed under a microscope, and cells contained two or more nuclei were identified as osteoclasts-like cells [17]. For bone resorption assay, the purified BMMNCs (2 × 10^5^ cells/well) were seeded in 96-well Osteo Assay Surface plates and inducted with M-CSF and RANKL. After being treated with MWCNTs (0, 1 and 5 μg/ml) for 12 days, the wells were washed with 10% sodium hypochlorite and rinsed with water for 3 times. Then the images of each well were captured using a microscope after the wells were sufficiently dried at room temperature. The bone absorption surface area was quantified by ImageJ software.

### Phenotypical analysis of macrophages

Raw264.7 was seeded in a 6-well plate and treated with MWCNTs (0, 1 and 5 μg/ml) for 7 days, then the total protein was extracted and quantified. According to previous work, the expression of iNOS and Arg-1 was detected by western blot [18]. Meanwhile, after 7 days of culture, the supernatants were collected for the detection of IL-4 and IL-6. The experiment was carried out according to the instructions of the kits.

### Co-culture of BMSCs and Raw264.7 cells

BMSCs and Raw264.7 were seeded into transwell 6-well plate (0.4 μm), of which BMSCs were seeded into upper chamber (1 × 10^7^ cells/well), and Raw264.7 cells were seeded into lower chamber (1 × 10^4^ cells/well). After 24 h, the medium in both upper and lower chambers was removed. Then, 1 ml of the complete medium with 10% osteogenic inducer containing different concentrations of MWCNTs (0, 1 and 5 μg/ml) was added in the upper chamber of each well. Meanwhile, 2 ml of the complete medium with different concentrations of MWCNTs (0, 1 and 5 μg/ml) was added in the lower chamber. A uniform solution was obtained by ultrasound just before the experiment. All the medium was changed every 2 days for 3 times. After 7 days, the culture medium of BMSCs and Raw264.7 cells was collected, and the supernatant was collected by centrifugation. IL-4 and IL-6 in the supernatant were tested according to the kits’ instructions [18].

In order to evaluate the effect of the osteogenic differentiation of BMSCs on the co-cultivation of two kinds of cells, the medium collected from Raw264.7 was mixed with the complete fresh medium, and was used for BMSCs culture [18]. After 7 days culture of Raw264.7 cells with different concentrations of MWCNTs, the supernatant was collected and obtained as the conditioned medium. Then, the specific medium for BMSCs was prepared by mixing fresh DMEM with above collected conditioned medium in a ratio of 1:1 and used for ALP staining, mineralization and osteogenic-related genes Runx2, OCN and ALP.

### Vascularization of HUVECs *in vitro*

Migration and angiogenesis of HUVECs were detected as published work and details were listed in the Supplementary Material. To illustrate the vascularization effects of HUVECs in the presence of BMSCs and Raw264.7 treated with MWCNTs, the conditioned medium collected from co-culture model of BMSCs and Raw264.7 was mixed with the fresh DMEM in a ratio of 1:2 to prepare HUVECs specific medium. HUVECs in this specific medium were used to mimic the migration and angiogenesis of HUVECs in the co-culture system. The details were listed in the Supplementary Material [19].

### Calvarial organ cultures

The calvaria of 5-day-old ICR sucklings was cut in half along the longitudinal axis and placed on a self-made stainless-steel scaffold in the 48-well plates containing DMEM complete medium with 2 mM glutamine. After 2 days, 100 nmol/l LPS and the calvaria were cultured for another 3 days. Then macrophages were seeded into the wells under the stainless-steel scaffold in the 48-well plate and cultured overnight. MWCNTs were added to the wells and cultured for 14 more days. Then, the calvaria was fixed in 4% paraformaldehyde at 4°C and scanned by μ-CT (SkyScan1172, Bruker, Belgium). CTvox built the bone morphology, and CTan calculated related bone parameters [20]. Moreover, the immunohistochemical staining of OCN and CTSK was operated after the decalcification of calvaria.

### Statistical analysis

All experiments were repeated 3 times independently, and the experimental results were expressed in the form of mean ± SEM. The student’s t-test analyzed the statistics of two groups, and the one-way ANOVA processed the statistics of three or more groups, in which *P < 0.05, **P < 0.01, ***P < 0.001 and #*P* < 0.05, ##*P* < 0.01, ###*P* < 0.001 indicated statistical differences.

## Results and discussion

### Characterizations of MWCNTs

**Figure 1.**
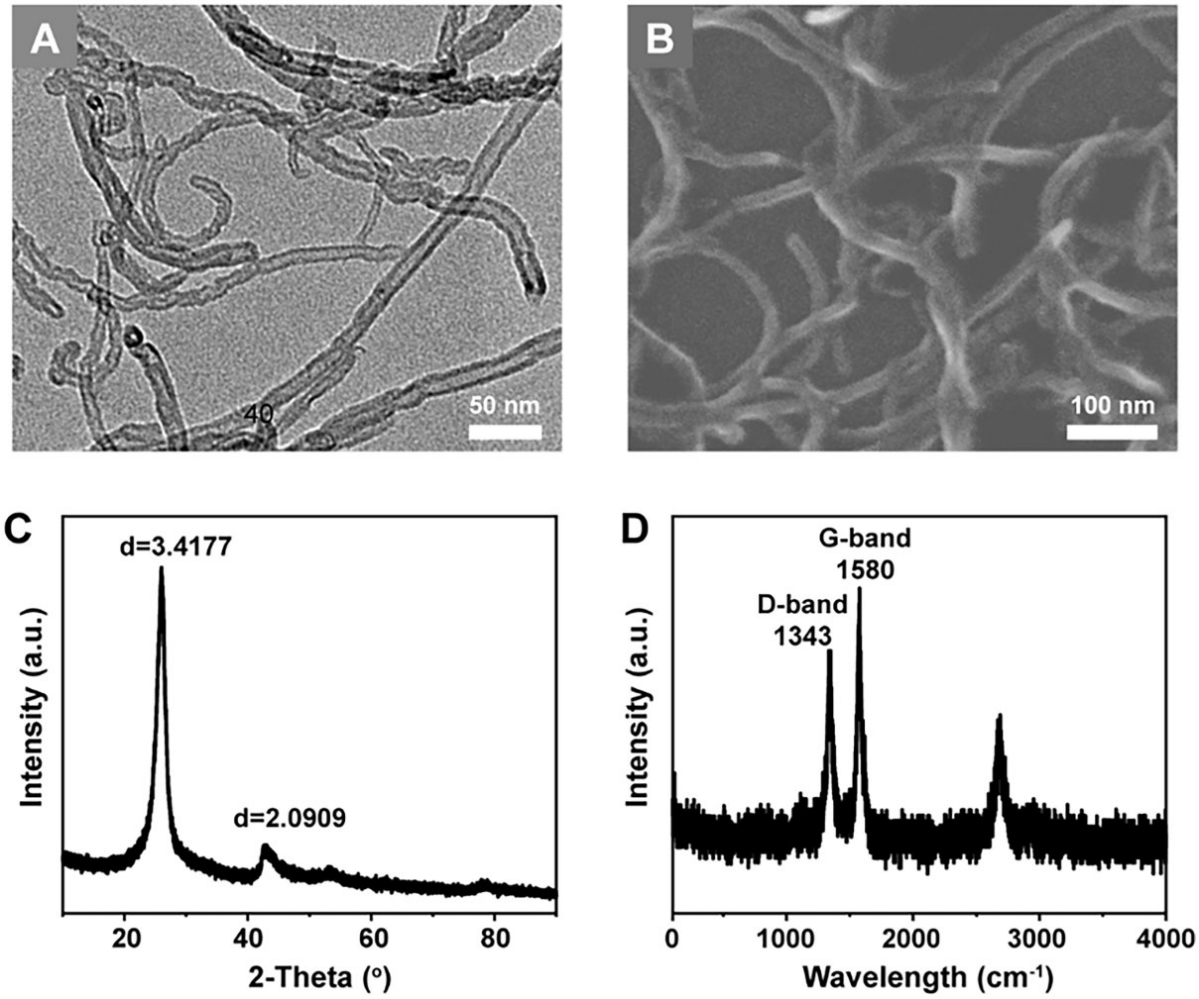
Characterizations of MWCNTs. (A) TEM images. (B) SEM images. (C) XRD spectrum. (D) Raman spectrum.

MWCNTs used in this work showed the tubular structure with the length of 20–30 μm and the diameter of 10–20 nm (Fig. 1A and B). The diameter sizes of MWCNTs were similar to the size of three strands of collagen, which might promote the mineralization [4]. The XRD patterns showed the crystallographic structure of the sample. The diffraction peak of 26.1° was assigned to the (002) refection of graphite (Fig. 1C). The Zeta potential of MWCNTs was about –28 mV, indicating the carboxylated surface and the good dispersion in water. Moreover, there were two characteristic peaks in the Raman spectrum, including the G peak near 1580 cm^−1^ and the D peak at 1343 cm^−1^ (Fig. 1D). The G peak reflected sp^2^ hybridization, a six-membered ring plane stretching and symmetrical vibration. The high-strength G peak reflected the high degree of graphitization of carbon materials [21]. The D peak caused by the edges or defects of the crystallite plane reflected the defect structure inside the materials. The peak height ratio of D and G peak *I*_d_/*I*_g_ was 0.807, which indicated that MWCNTs had a higher degree of graphitization and a higher proportion of ordered structures [21, 22]. The high degree of graphitization of MWCNTs benefited the bio-safety of this material [23].

### The osteogenic differentiation of BMSCs and MC3T3-E1

**Figure 2.**
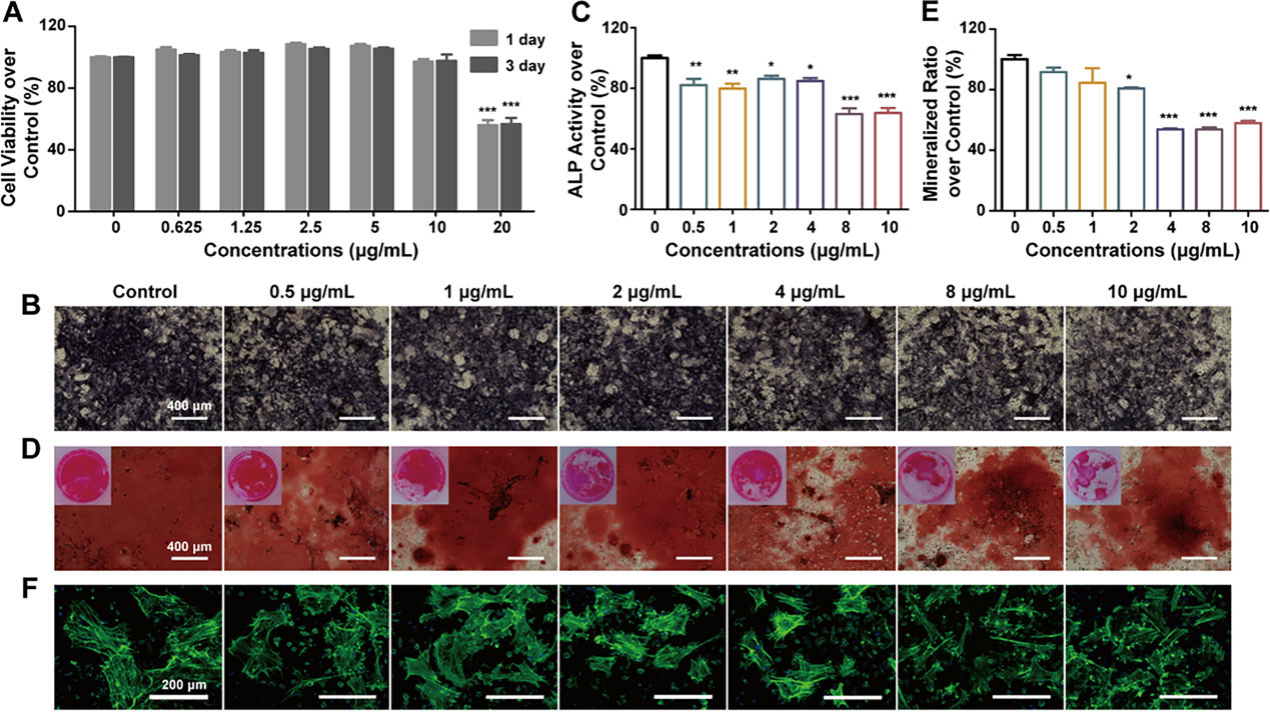
Osteogenic effects of MWCNTs on BMSCs. (A) BMSCs cell viability after being treated with different concentrations of MWCNTs for 1 and 3 days. ALP expression (B) and ALP activity (C) after being treated with MWCNTs for seven days. Mineralized nodules (D) and quantity analysis (E) of alizarin red staining after being treated with MWCNTs for 21 days. (F) Actin microfilaments of BMSCs after being treated with MWCNTs for seven days. *P < 0.05, **P < 0.01 and ***P < 0.001 represent the comparison with the control group.

BMSCs and MC3T3-E1 are two osteogenic cells derived from bone tissue. BMSCs and MC3T3-E1 can secrete minerals during osteogenesis and repair the damaged bone tissue [24]. The osteogenic effects of MWCNTs were firstly evaluated on BMSCs. When treated with MWCNTs at the concentration of 10 μg/ml or below, the cell viabilities of BMSCs kept nearly 100%. However, the cell viability was reduced to 60% when the concentration of MWCNTs was 20 μg/ml (Fig. 2A). Therefore, the concentration of MWCNTs in the following test for BMSCs was all below 20 μg/ml. ALP is the earliest marker for BMSCs osteogenic differentiation. ALP activity is considered an indicator of the osteogenic differentiation of BMSCs and the index of osteoblasts. After treating with MWCNTs for 7 days, the dark purple substances of ALP staining showed lower in the 8 and 10 μg/ml treatment groups (Fig. 2B) and the activity of ALP was decreased in all MWCNTs treatment groups (Fig. 2C). Meanwhile, the osteogenic-related genes OCN, RUNX2 and OPG were down-regulated in MWCNTs treatment groups (Supplementary Fig. S1A–C). Mineralization occurs in the late stage of osteogenic differentiation. The tissue is incorporated into the secreted matrix and followed by the deposition of calcium salts. Subsequently, the matrix is mineralized and formed opaque areas [25]. Therefore, the number of mineralized nodules indicates the amount of calcium deposition. With the treatment of 8 and 10 μg/ml MWCNTs, the mineralization of BMSCs was lower than the control group (Fig. 2D). Furthermore, the mineralization ratios of BMSCs generally decreased with the increasing concentrations of MWCNTs (Fig. 2E).

The cytoskeleton maintains the morphology of cells, which is closely related to the osteogenic differentiation of BMSCs. In addition, the rapid recruitment of bone cells also greatly affects the process of bone repair [12]. Therefore, the actin structure and migration ability of BMSCs can also reflect the bone-forming effects. Cells in the control group showed better diffusion, and had a well-organized cytoskeleton network with more adherent cells and more obvious filopodia protruding from actin staining (Fig. 2D). There was no obvious inhibition to the intracellular microfilament structures when the concentrations of MWCNTs were 0.5, 1, 2 and 4 μg/ml. However, with the treatment of 8 and 10 μg/ml MWCNTs, the expression of actin and the extension of pseudopodia were significantly inhibited (Fig. 2F). Meanwhile, the migration of BMSCs was inhibited in the presence of 5 μg/ml MWCNTs compared with the control group (Supplementary Fig. S1D). Therefore, 1 and 5 μg/ml MWCNTs were used in the following experiments.

The osteogenic effects of MWCNTs were further evaluated on osteoblasts MC3T3-E1. The cell viability of MC3T3-E1 was still above 80% after being treated with 10 μg/ml MWCNTs for 3 days (Supplementary Fig. S2A). With the treatment of 8 μg/ml MWCNTs for 7 days, the activity and expression of ALP in MC3T3-E1 cells were significantly inhibited (Supplementary Fig. S2B and C). Furthermore, the osteogenic-related genes Runx2, OCN and OPG were down-regulated at various degrees with 1 and 5 μg/mL MWCNTs (Supplementary Fig. S2D–F).

### The osteoclasts differentiation of BMMNCs

**Figure 3.**
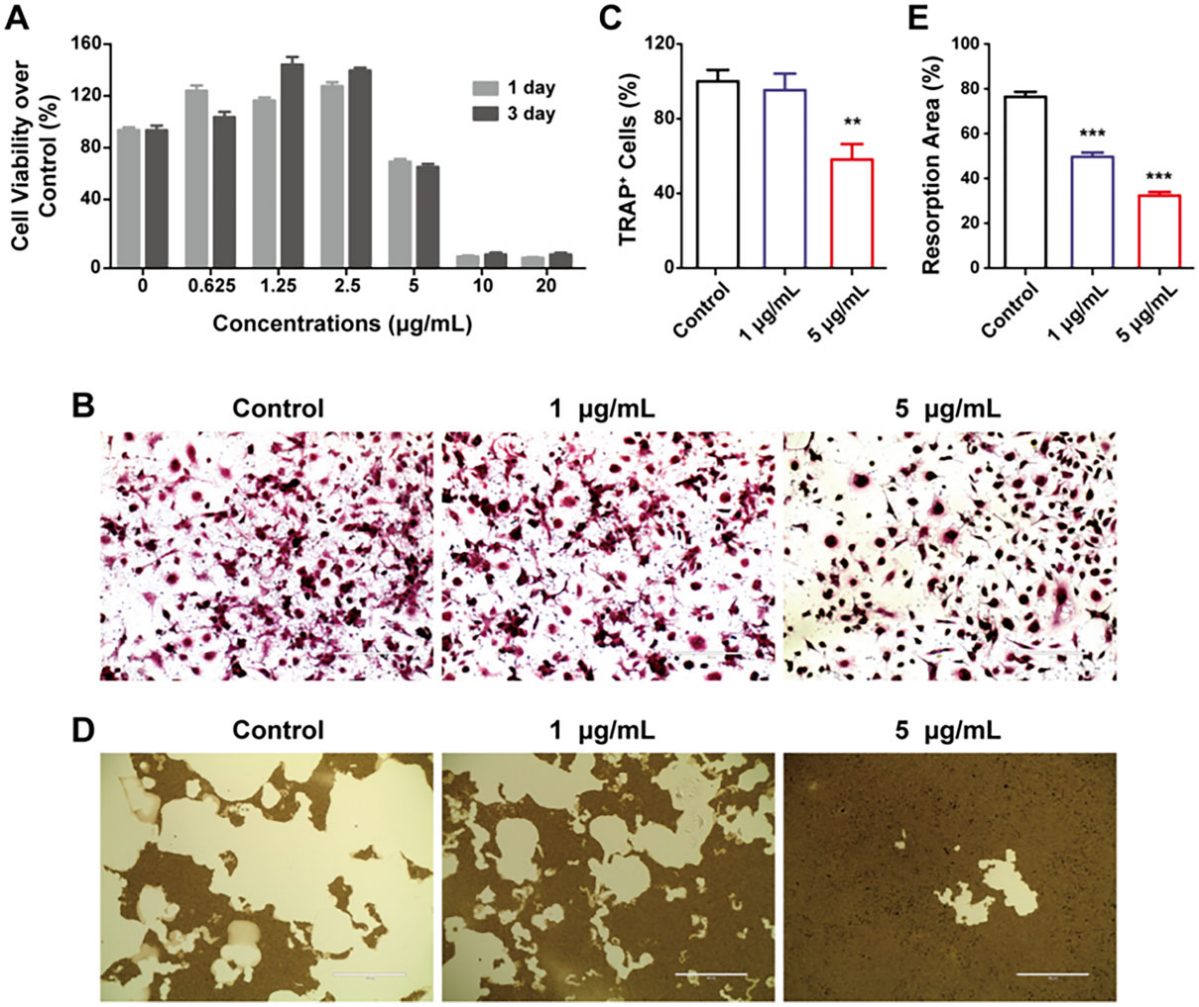
The osteoclasts differentiation effects of MWCNTs on BMMNCs. (A) The viability of BMMNCs treated with MWCNTs for 1 and 3 days. (B) TRAP staining of BMMNCs at M-CSF and RANKL co-stimulation for 7 days. (C) The counts of TRAP-positive cells were quantified from TRAP staining results. (D) The pit formation in the osteo assay surface plate at M-CSF and RANKL co-stimulation for seven days. (E) The bone resorption area is quantified from (D). **P < 0.01 and ***P < 0.001 represent the comparison with the control group.

The osteoclasts derived from BMMNCs regulate the remodeling process of bone in cooperation with osteoblasts. The proliferations of BMMNCs were promoted by MWCNTs when the concentrations were 2.5 μg/ml or lower. However, when the concentration of MWCNTs were 5 μg/ml or above, the cell viabilities of BMMNCs were suppressed greatly (Fig. 3A). Moreover, the generation of TRAP positive osteoclasts-like cells was significantly inhibited with the treatment of 5 μg/mL MWCNTs (Fig. 3B and C). At the same concentration of MWCNTs, the bone resorption capacity of the osteoclasts-like cells was significantly inhibited on the pit formation in the Osteo Assay Surface plate (Fig. 3D and E). Although the formation of TRAP-positive osteoclasts-like cells in the presence of 1 μg/ml MWCNTs group was comparable to the control group, the bone resorption capacity greatly decreased in the pit formation. The results indicated that MWCNTs could inhibit the osteoclast differentiation and maturation of BMMNCs, confirming the inhibition of MWCNTs to bone resorption *in vivo* [26].

### The effects of macrophage polarization on Raw264.7

**Figure 4.**
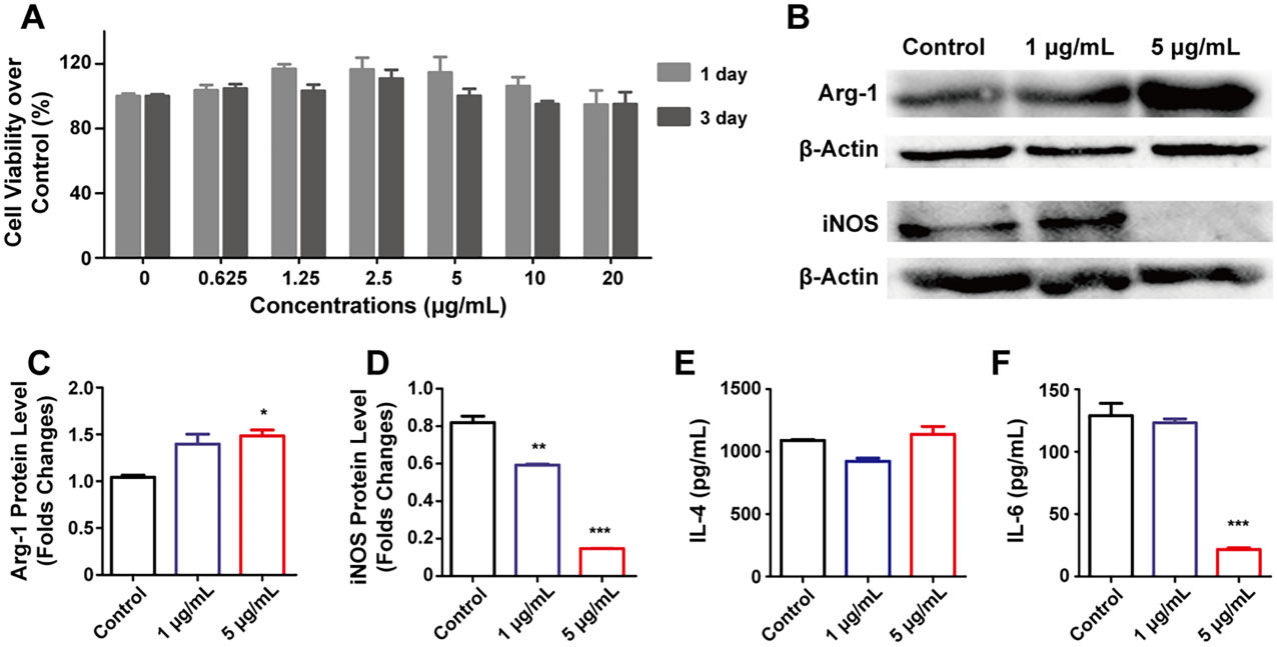
Cell viability and immunophenotype of Raw264.7 cells treated with MWCNTs. (A) The viability of Raw264.7 cells treated with MWCNTs for 1 and 3 days. (B) Arg-1 and iNOS protein expression of macrophages detected by Western blot with the treatment of MWCNTs for 7 days. The relative quantification of arg-1 (C) and iNOS (D) protein from (B) the secretion cytokines of macrophages, IL-4 (E) and IL-6 (F), were detected by ELISA after being treated with MWCNTs for 7 days. *P < 0.05, **P < 0.01 and ***P < 0.001 represent the comparison with the control group.

Among the heterogeneous cells in bone marrow, macrophages play a key role to fight against infection and inflammation. Macrophages are classified into M1 type and M2 type, and M2 macrophages promote bone formation [27]. Dong *et al.* [28] found that macrophages can polarize to M1 and M2 types through the STAT and IRF signaling pathways when the lungs were exposed to MWCNTs *in vivo*. Nevertheless, different polarization types of macrophages show pro-inflammatory or anti-inflammatory effects on osteogenic differentiation. Herein, we investigate the macrophage polarization induced by MWCNTs in the bone microenvironment. MWCNTs displayed no obvious cytotoxicity towards Raw264.7 cells for 1 and 3 days at the concentrations of 0.625–20 μg/ml (Fig. 4A). On the other hand, the M2-immunophenotype factor of macrophages, Arg-1, was upregulated with 1 and 5 μg/ml MWCNTs (Fig. 4B and C). M2-type transformation of macrophages means less secretion of M1-type cytokines. In addition, the expression of iNOS was lower in the MWCNTs treatment groups than that of the control group (Fig. 4B and D). Meanwhile, although there was no statistic significant difference in the secretion of IL-4 in all the groups, this cytokine was slightly higher with 5 μg/ml MWCNTs (Fig. 4E). Additionally, the secretion of IL-6 was inhibited greatly with 5 μg/ml MWCNTs (Fig. 4F). Moreover, obvious M2 polarization of the macrophages was observed with the treatment of MWCNTs at 5 μg/ml but not at 1 μg/ml. IL-4 in bone marrow stroma maintains the stemness of BMSCs in the early stage of bone repair and accelerates cell migration and proliferation [29]. Therefore, when the osteogenic effects of BMSCs and osteoblasts were inhibited by MWCNTs (Fig. 2 and Supplementary Fig. S2), it is necessary to reveal the osteogenic effects with M2 polarization macrophages.

### The osteogenic differentiation of co-culture of BMSCs and Raw264.7

**Figure 5.**
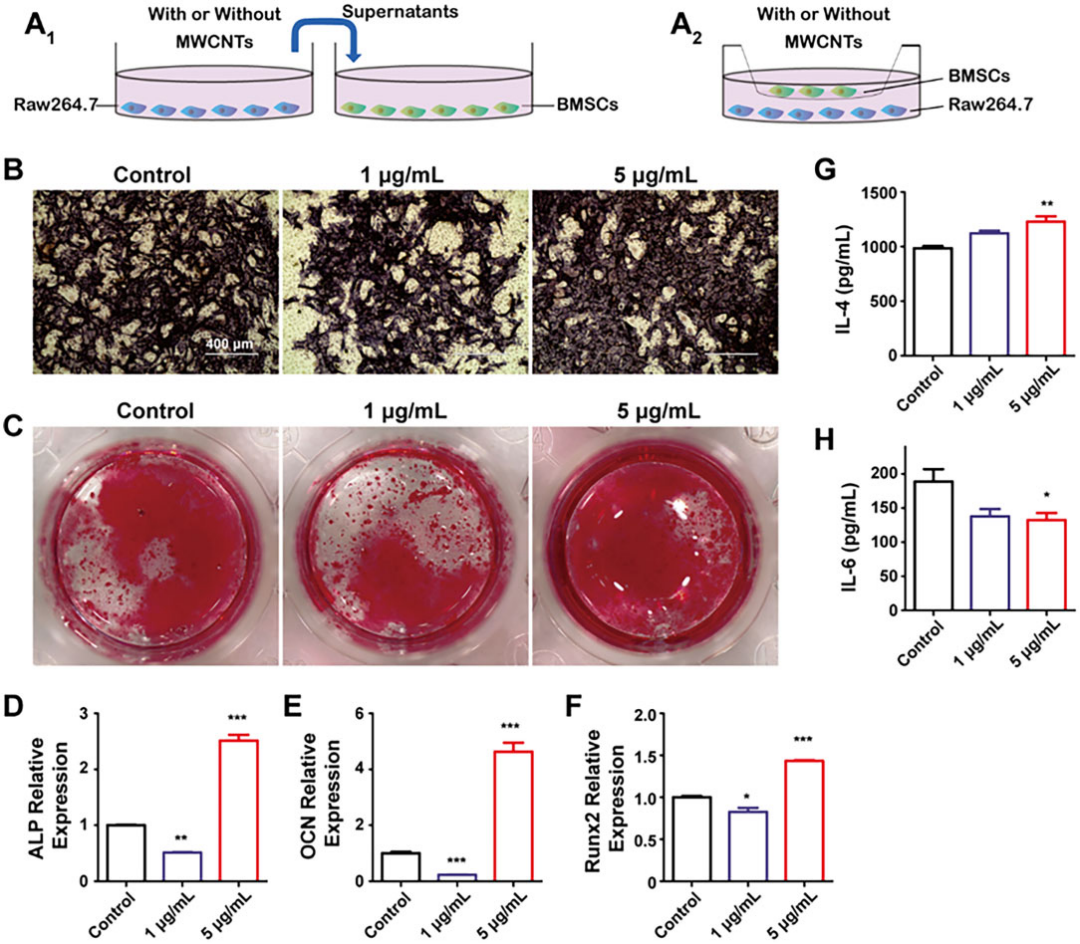
Effects of MWCNTs on the co-culture of BMSCs and Raw264.7 cells. The scheme of BMSCs-Raw264.7 co-culture system for osteogenesis detection (A_1_) and polarization detection of macrophages (A_2_). ALP (B) and alizarin red (C) staining of BMSCs co-cultured with Raw264.7. The mRNA expression of osteogenic-related genes ALP (D), OCN (E) and Runx2 (F) of BMSCs co-cultured with Raw264.7. IL-4 (G) and IL-6 (H) secretion in BMSCs–Raw264.7 co-culture system for macrophage polarization. *P < 0.05, **P < 0.01 and ***P < 0.001 represent the comparison with the control group.

The osteogenic behavior at the single-cell level cannot sufficiently illustrate the function of MWCNTs due to the lack of the signal transducitons among different types of cells. Co-cultivation of BMSCs and macrophages provides a good way to study their interaction [30–32]. In the BMSCs–Raw264.7 co-culture system for osteogenesis detection (Fig. 5A_1_), with the treatment of 5 μg/ml MWCNTs, the qualitative and quantitative results of ALP, the mineralization and the expression of osteogenic-related genes ALP, OCN and Runx2 were all higher than those in control group (Fig. 5B–F), which was consistent with the M2 polarization macrophages (Fig. 4). However, in the presence of 1 μg/ml MWCNTs, the osteogenic differentiation and mineralization of BMSCs were still inhibited, which could be related to the non-polarization of macrophages (Figs 4 and 5B–F). Meanwhile, in the BMSCs–Raw264.7 co-culture system (Fig. 5A_2_), there was no significant difference in the secretion of IL-4 and IL-6 with 1 μg/ml MWCNTs compared with control group. However, the secretion of IL-4 was increased and the secretion of IL-6 was decreased with 5 μg/ml MWCNTs (Fig. 5G and H). When treated with MWCNTs, osteogenic differentiation of BMSCs can be inhibited. Nevertheless, M2 polarization macrophages induced by 5 μg/ml MWCNTs can enhance osteogenic differentiation of BMSCs. Therefore, the interaction between BMSCs and macrophages played an important role in the osteogenic differentiation when treated with MWCNTs.

### The bone formation effects on co-culture of calvaria and macrophages

**Figure 6.**
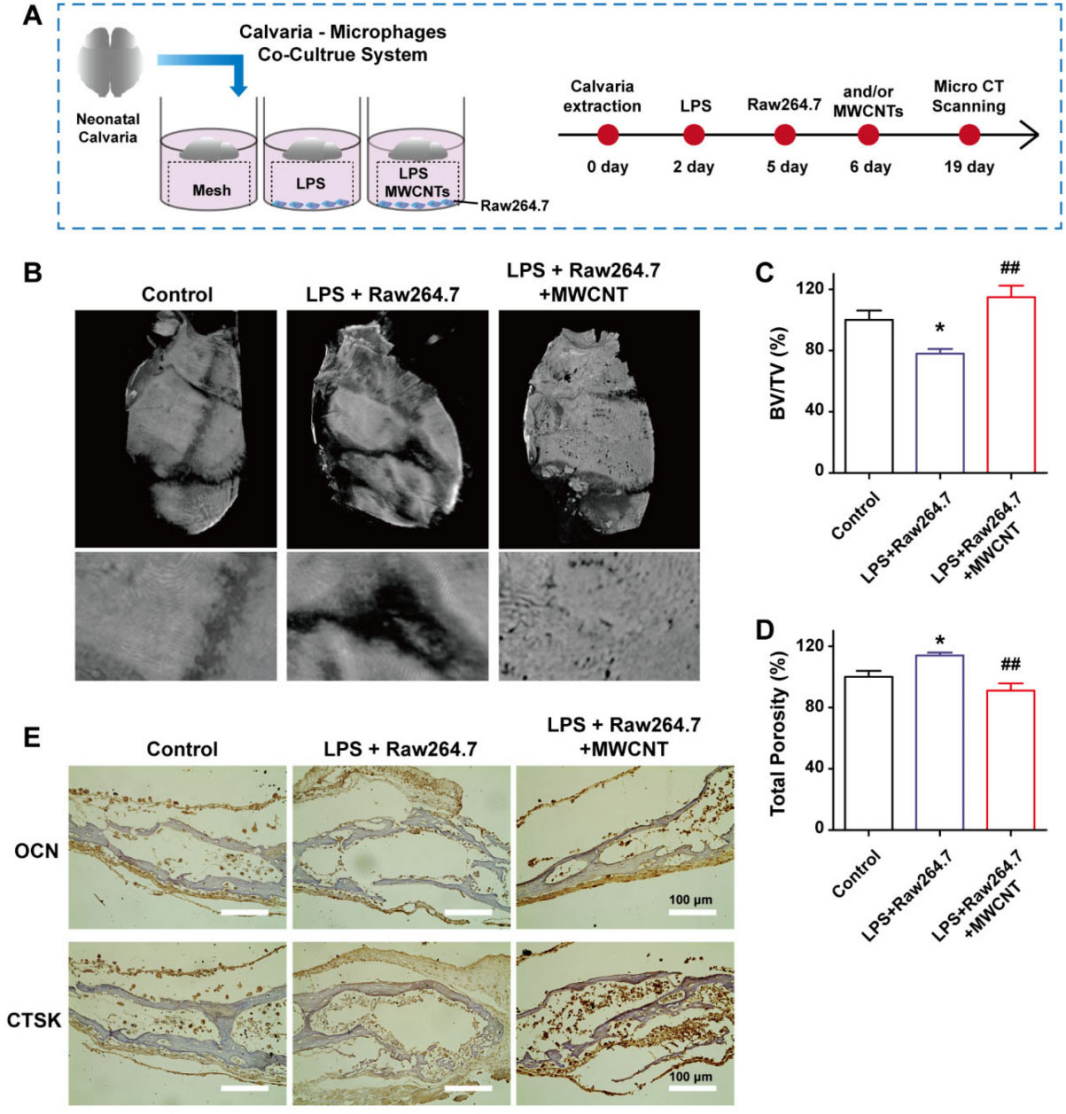
Effect of MWCNTs on bone formation in the co-culture of calvaria and macrophages. (A) The neonatal calvaria–Raw264.7 co-culture system and the flow chart scheme. (B) The whole and part of micro-CT images of calvaria cultured with MWCNTs for 14 days. Bone volume fraction (C) and total porosity (D) from CT images. (E) Immunohistochemical staining analysis of OCN and CTSK in the calvaria slice. *P < 0.05 represents the comparison with the control group; ##*P* < 0.01 indicates the comparison with the Raw264.7 group.

The neonatal calvaria is rich in osteoblasts and osteoclasts, therefore, it is a favorable *ex vivo* culture model for bone remodeling, especially to simulate cell–cell contact within the bone microenvironment [33]. Here, neonatal calvaria and Raw264.7 co-culture systems were employed to investigate the bone formation regulated by M2-polarized macrophages after the treatment of MWCNTs (Fig. 6A). This co-culture system induced by LPS was used as the bone defects model and operated as shown in Fig. 6A [34]. In the LPS + Raw264.7 group, the calvaria showed an obvious bone loss from CT images (Fig. 6B) and CT parameters analysis (Fig. 6C and D). When treated with MWCNTs, calvaria’s morphology and CT parameters showed distinct improvement compared with the LPS + Raw264.7 group, which showed almost no significant difference with control group (Fig. 6B–D). Meanwhile, OCN in the calvaria derived from osteoblasts was detected by immunohistochemical staining. The LPS + Raw264.7 + MWCNTs group showed the highest OCN expression, but almost no changes were detected in control group and LPS + Raw264.7 group (Fig. 6E). In contrast, CTSK showed higher expression in the LPS + Raw264.7 group than the LPS + Raw264.7 + MWCNTs group (Fig. 6E). It was confirmed that MWCNTs might improve bone formation by inducing M2 polarization of macrophages and inhibiting osteoclasts differentiation.

### The angiogenesis of HUVECs

**Figure 7.**
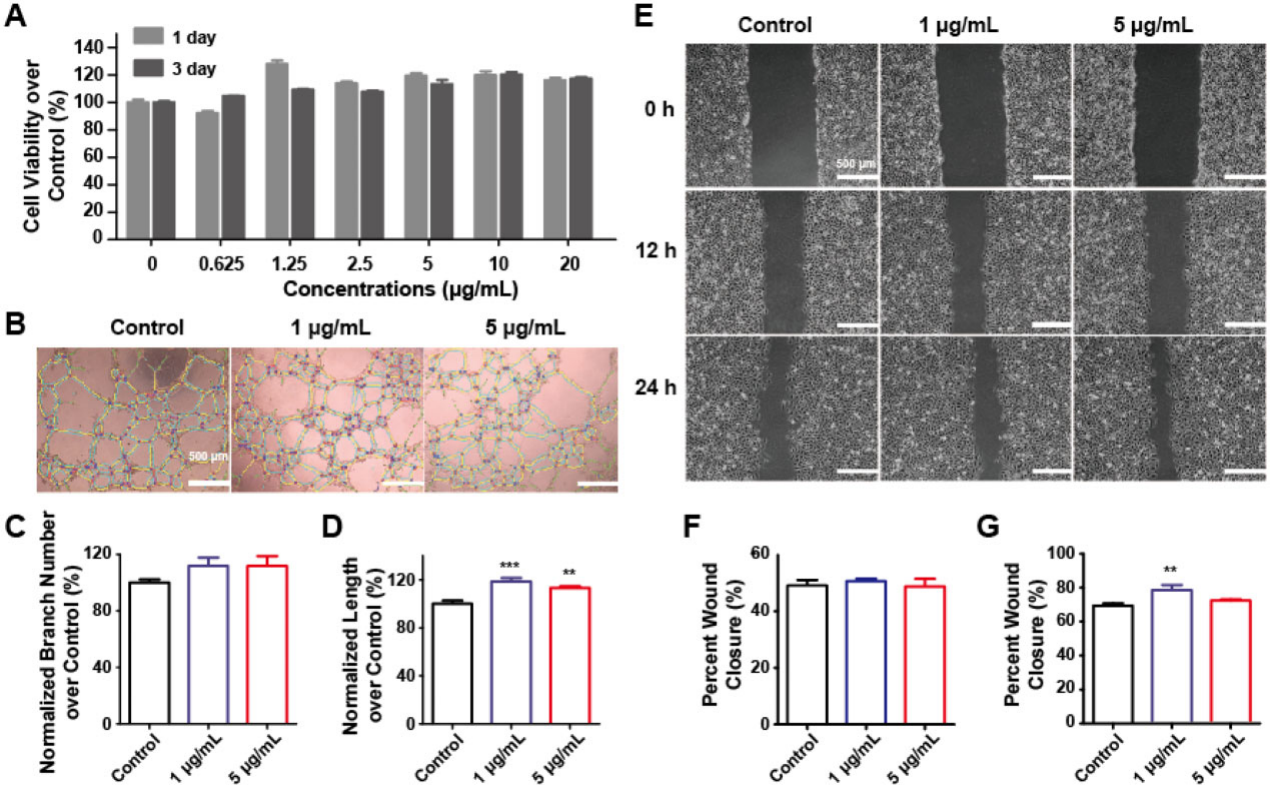
The angiogenesis of MWCNTs on HUVECs. (A) Cell viability treated with different concentrations of MWCNTs for 1 and 3 days. (B) The capillary-like network of HUVECs was treated with 1 and 5 μg/ml MWCNTs. The number of branches (C) and total length (D) of B were quantified by ImageJ software. (E) The migration abilities of HUVECs with MWCNTs were tested by wound healing assay. The quantitative results of HUVECs migration at 12 h (F) and 24 h (G). **P < 0.01 and ***P < 0.001 represent the comparison with the control group.

Since bones are highly vascularized organs, angiogenesis plays an important role in osteogenesis. Therefore, the angiogenesis of MWCNTs was evaluated on vascular endothelial cells. First of all, MWCNTs had no obvious cytotoxicity in HUVECs at the concentrations of 0.625–20 μg/ml (Fig. 7A). Moreover, compared with the control group, 1 and 5 μg/ml MWCNTs promoted the self-assembling and elongation of HUVECs as indicated from the number of branches and the length of blood vessels of the capillary-like network (Fig. 7B–D). Meanwhile, as indicated from the wound healing assay, the migration of endothelial cells, which is associated with angiogenesis, was also enhanced in the presence of 1 and 5 μg/ml MWCNTs (Fig. 7E–G).

Researchers have found that the angiogenesis of HUVECs can be weakened in the osteogenesis stage of BMSCs [35, 36]. Based on the osteogenic differentiation of BMSCs and the M2-polarization of macrophages induced by 5 μg/ml MWCNTs (Fig. 5B–F), BMSCs–Raw264.7–HUVECs co-culture system was employed to evaluate the vascularization (Supplementary Fig. S3A). Compared with the control group, the number of branches and the length of blood vessel were significantly elevated by 1 μg/ml MWCNTs but were decreased by 5 μg/ml MWCNTs (Supplementary Fig. S3B–D). These results indicated that 5 μg/ml MWCNTs inhibited the angiogenesis ability of endothelial cells in the BMSCs–Raw264.7–HUVECs co-culture system. Further, the scratching experiment revealed that MWCNTs didn’t promote the migration of endothelial cells (Supplementary Fig. S3D–F). The contradictory effects of MWCNTs on angiogenesis revealed the inconsistence of the single-cell level experiment the cell–cell interaction model, which should be thoroughly investigated *in vivo* in the future.

## Conclusion

In this work, the bone formation effects of MWCNTs were investigated *in vitro* and *ex vivo*, and both single-type cells experiment and co-culture models of different types of cells were established. The carboxylated MWCNTs with a length of 20–30 μm and a diameter of 10–20 nm were used because of the similar sizes and structures with collagen. MWCNTs inhibited the osteogenic differentiation of both BMSCs and osteoblasts MC3T3-E1 when treating these two types of cells independently. Similarly, MWCNTs also inhibited the cell proliferation and the osteoclasts differentiation of BMMNCs. In addition, MWCNTs was found to induce M2-polarizaiton of macrophages and to promote the angiogenesis of HUVECs. Interestingly, in the co-culture of BMSCs and macrophages, MWCNTs promote the osteogenic differentiation of BMSCs and the bone formation in the calvaria *ex vivo*. These results revealed the inconsistence of the single-cell level experiment the cell–cell interaction model, which should be thoroughly investigated *in vivo* in the future. We proposed that the MWCNTs stimulate Raw264.7 to M2 type macrophages, which secreted IL-4 and showed anti-inflammatory effects on BMSCs to promote bone formation. This work focuses on the basic investigation of MWCNTs on bone, and will be helpful for developing MWCNTs-based bone repair materials in the future.

The detailed methods for the dispersion and chemical characterization of MWCNTs, the culture and cell viability assay, ALP, actin and mineralization staining and quantitative test, osteogenesis-related genes detection, the migration and angiogenesis were listed in the supplementary Material. Meanwhile, osteogenic-related genes expression and cell migration of BMSCs, the effects of MWCNTs on MC3T3-E1 cells and the angiogenesis of MWCNTs on the co-culture of HUVECs and BMSCs-Raw264.7 also are supplied in Supplementary Figs S1–S3.

## Supplementary Material

rbad042_Supplementary_Data
