# Supplementary material for: Multi-walled carbon nanotubes reversing the bone formation of bone marrow stromal cells by activating M2 macrophage polarization
Source: Regen Biomater. 2023 Apr 22;10:rbad042. doi: 10.1093/rb/rbad042 (PMC10234760; doi:10.1093/rb/rbad042)
Supplement: rbad042_Supplementary_Data [file rbad042_supplementary_data.docx]

Supporting Information

**Multi-walled carbon nanotubes** **reversing the bone formation of bone marrow stromal cells by** **activating M2 macrophage polarization**

Runlian Lin ^1^, Kun Ge ^1,^*, Dehui Fan ^1^, Jing Li ^1^, Guoqiang Zhou ^1,2^, Kaihan Zhang ^3^, Yuanyu Huang ^4^, Lili Ma ^1,^*, Jinchao Zhang ^1^

^1^ Key Laboratory of Medicinal Chemistry and Molecular Diagnosis of the Ministry of Education, Key Laboratory of Chemical Biology of Hebei Province, College of Chemistry and Material Science, Hebei University, Baoding, 071002, China

^2^ College of Basic Medical Science, Hebei University, Baoding, 071000, China

^3^ Department of Chemistry, The University of Manchester, Manchester, M13 9PL, U.K.

^4^ School of Life Science, School of Medical Technology, Advanced Research Institute of Multidisciplinary Science, Key Laboratory of Molecular Medicine and Biotherapy, Key Laboratory of Medical Molecule Science and Pharmaceutics Engineering, Beijing Institute of Technology, Beijing, 100081, China

* Corresponding author. Key Laboratory of Medicinal Chemistry and Molecular Diagnosis of the Ministry of Education, Key Laboratory of Chemical Biology of Hebei Province, College of Chemistry and Environmental Science, Hebei University, Baoding, 071002, China

E-mail addresses: [gekun@hbu.edu.cn](mailto:gekun@hbu.edu.cn) (K. Ge), [hbumary@163.com](mailto:hbumary@163.com) (L. Ma).

**Dispersion and characterizations of multi-walled carbon nanotubes (MWCNTs)**

MWCNTs (10 mg) and dispersant (2 mg) were suspended in deionized water (9.988 mL) and dispersed by ultrasound for 30 min. Then, MWCNTs dispersions were centrifuged (2000 rpm, 30 min) to obtain precipitation. Finally, MWCNTs precipitation was resuspended in deionized water and stored in a dark and dry place for use.

The content of MWCNTs in the dispersions was analyzed by a thermogravimetric analyzer (NETZSCH STA449C, Germany). The morphology and size of obtained MWCNTs were characterized by TEM (FEI Tecnai G2 F20 S-TWIN, America) and SEM (JEOL JSM-7500F, Japan). In addition, the characteristic structure of MWCNTs was detected by Raman (Renishaw inVia, Britain).

**Isolation and culture of bone marrow stromal cells (BMSCs)**

The primary BMSCs were obtained from 4-6 weeks of ICR female mice. BMSCs were cultured in the DMEM containing 10% FBS at normal cell culture conditions. The extracted BMSCs were used for follow-up tests after 3 days [1].

**Isolation and culture of bone marrow mononuclear cells (BMMNCs)**

The primary BMMNCs were extracted from the leg bones of female ICR mice of 8-10 weeks. First, all the cells from the bone were cultured in DMEM containing 15% FBS and 1% penicillin/streptomycin for 16 h. Next, nonadherent cells were collected and cultured in complete DMEM with 30 ng/mL M-CSF for 3 days. Then BMMNCs were cultured in complete DMEM medium with 30 ng/mL M-CSF and 50 ng/mL RANKL for the following experiments.

**Culture of MC3T3-E1, Raw264.7, and human umbilical vein endothelial cells (HUVECs)**

The osteoblasts MC3T3-E1, macrophages Raw264.7, and HUVECs were cultured in ɑ-MEM, DMEM, and high-sugar DMEM (HDMEM) medium containing 10% FBS and 1% penicillin/streptomycin, respectively.

**Cell viability assay**

CCK8 kit was employed for BMSCs and BMMNCs viability test after treatment with different concentrations of MWCNTs [2,3]. BMSCs or BMMNCs were seeded in a 96-well plate. After 24 h (BMMNCs) or 72 h (BMMNCs), 10 μL CCK8 solution was added into each well and incubated for another 2 h. Then the absorbance at 450 nm was measured with a multifunctional microplate reader (Molecular Devices, VersaMax, USA).

MTT assay was used for cell viability test for MC3T3-E1, Raw264.7, and HUVECs with treatment of different concentrations of MWCNTs [4]. After culturing the cells for 24 h and 72 h, 10 μL MTT (5 mg/mL) solutions were added to each well and incubated for another 4 h. Then the culture medium was discarded, and 100 μL DMSO was added to each well. The absorbance at 570 nm was measured with a multifunctional microplate reader.

The cell viabilities treated with MWCNTs were calculated by GraphPad.

**ALP staining**

BMSCs or MC3T3-E1 cells were cultured in a complete medium containing osteogenic inducer in 48-well plates and treated with different concentrations of MWCNTs. The cells were cultured for 7 days and the cell culture medium were refreshed every 1 or 2 days. Then, ALP was stained by BCIP/NBT alkaline phosphatase color development kit, and the images were recorded by a microscope (EVOS FL AUTO, ThermoFisher Scientific) [5].

**ALP quantitative**

BMSCs or MC3T3-E1 cells were cultured and treated as above. After 7 days of culture, the cells were lysed in Triton-X100 solution. Then, the ALP activity was measured by the alkaline phosphatase assay kit according to the manufacturer’s protocol and normalized with the total protein [6]. The data were analyzed by GraphPad.

**Actin staining**

BMSCs were cultured and treated as above. After 7 days, the cytoskeleton was stained with ActinGreen 488 ReadyProbes reagent in the dark for 30 min and observed under a microscope [4].

**Mineralization staining and quantification**

BMSCs were cultured and treated as above. After 21 days, the mineralized nodules were stained with Alizarin Red staining solution (100 μL, 0.1%) for 30 min and observed under a microscope. Then, the mineralized nodules were dissolved in a 10% cetylpyridinium chloride solution, and the absorbance at 570 nm was recorded by a multifunctional microplate reader [4]. The data were analyzed by GraphPad.

**BMSCs mobility assay**

BMSCs were seeded in 6**-**well plates and cultured with 0, 1, and 5 μg/mL MWCNTs for 48 h. Then, BMSCs were suspended to get the single-cell suspension in each group. Next, each upper chamber of 24-well transwell plates was coated with 80 μL diluted matrigel (diluted with the medium as 1:10) and BMSCs suspensions (2×10^4^ cells with 1% serum in the medium). Next, 500 μL complete medium was added to each lower chamber. After 24 h, the upper chambers were removed and fixed with 4% paraformaldehyde for 30 min, and were stained with crystal violet for 15 min. Later, the outside cells of the upper chambers were observed under a microscope [6].

**PCR detection of osteogenesis-related genes**

The expression of osteogenesis-related genes of BMSCs and MC3T3-E1 were detected by real-time PCR, including ALP, Runx2, OCN, and OPG (Table S1). The cells were cultured in 6-well plates and treated with 0, 1, and 5 μg/mL MWCNTs for 7 days. The total RNA from BMSCs were extracted by Trizol methods, and 1 μg of total RNA was used for the reverse transcription [7]. PCR reaction mixture was prepared according to the manufacturer’s protocol of the qPCR kit. The reaction conditions were 95 ^o^C degeneration for 5 min, 40 cycles for amplification (95 ^o^C degeneration for 30 sec, 60 ^o^C anneal for 30 sec, 72 ^o^C extending for 10 sec) and 72 ^o^C extending for 5 min. Furthermore, the melt curve process was set according to the instrument's default (StepOne Plus, ABI, USA).

Table S1. Primers of osteogenesis-related genes

| Primer Name | Sequences |
| --- | --- |
| Runx2 | F-TTCTCCAACCCACGAATGCAC  R-CAGGTACGTGTGGTAGTGAGT |
| OCN | 1. GAACAGACTCCGGCGCTA   R-AGGGAGGATCAAGTCCCG |
| OPG | F-AGTCCGTGAAGCAGGAGTG  R-CCATCTGGACATTTTTTGCAAA |
| ALP | F-GTTGCAAGCTGGGAAGAACAC  R-CCCACCCCGCTATTCCAAAC |
| GAPDH | F-GACTTCAACAGCAACTCCCAC  R-TCCACCACCCTGTTGCTGTA |

**Migration of HUVECs**

HUVECs were first seeded in a 6-well plate in complete cell culture medium with the density of 2×10^5^ cells/well. After 24 h, the medium was replaced by a fresh medium without FBS for starvation for 24 h to normalize all cells. Then the single-line wound was made with a 1 mL pipette tip. Meanwhile, HUVECs were added with different concentrations of MWCNTs. The migration of HUVECs in the wound area was observed and recorded at the same position at 0, 12, and 24 h, respectively [8].

**Angiogenesis of HUVECs**

HUVECs were cultured and starved as above. Then different concentrations of MWCNTs were mixed with matrigel at a volume ratio of 1:1 and incubated in the 48-well plate for 1 h at 37 ^o^C. After that, HUVECs were collected and seeded on the mixed matrix with the density of 1×10^5^ cells/well and incubated for 8 h. The length of the tubules and the number of branches were calculated by ImageJ software [8].


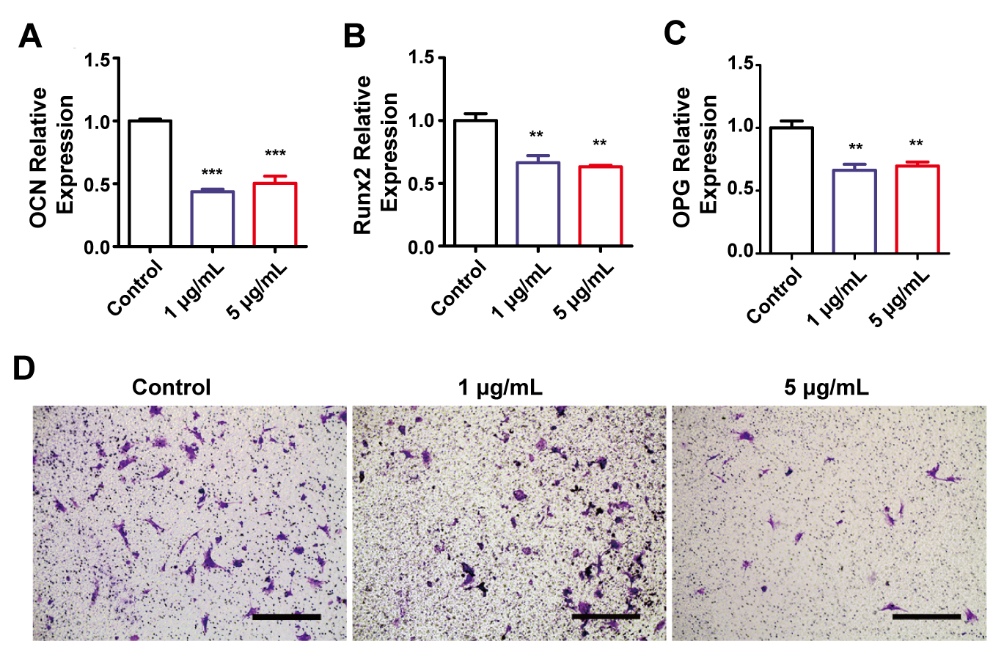


**Figure** **S1.** Osteogenic-related gene expression and cell migration of BMSCs. The mRNA expression of OCN (A), Runx2 (B), and OPG (C). (D) Cell migration of BMSCs after the treatment of MWCNTs for seven days. **P<0.01 and ***P<0.001 represent the comparison with the control group.


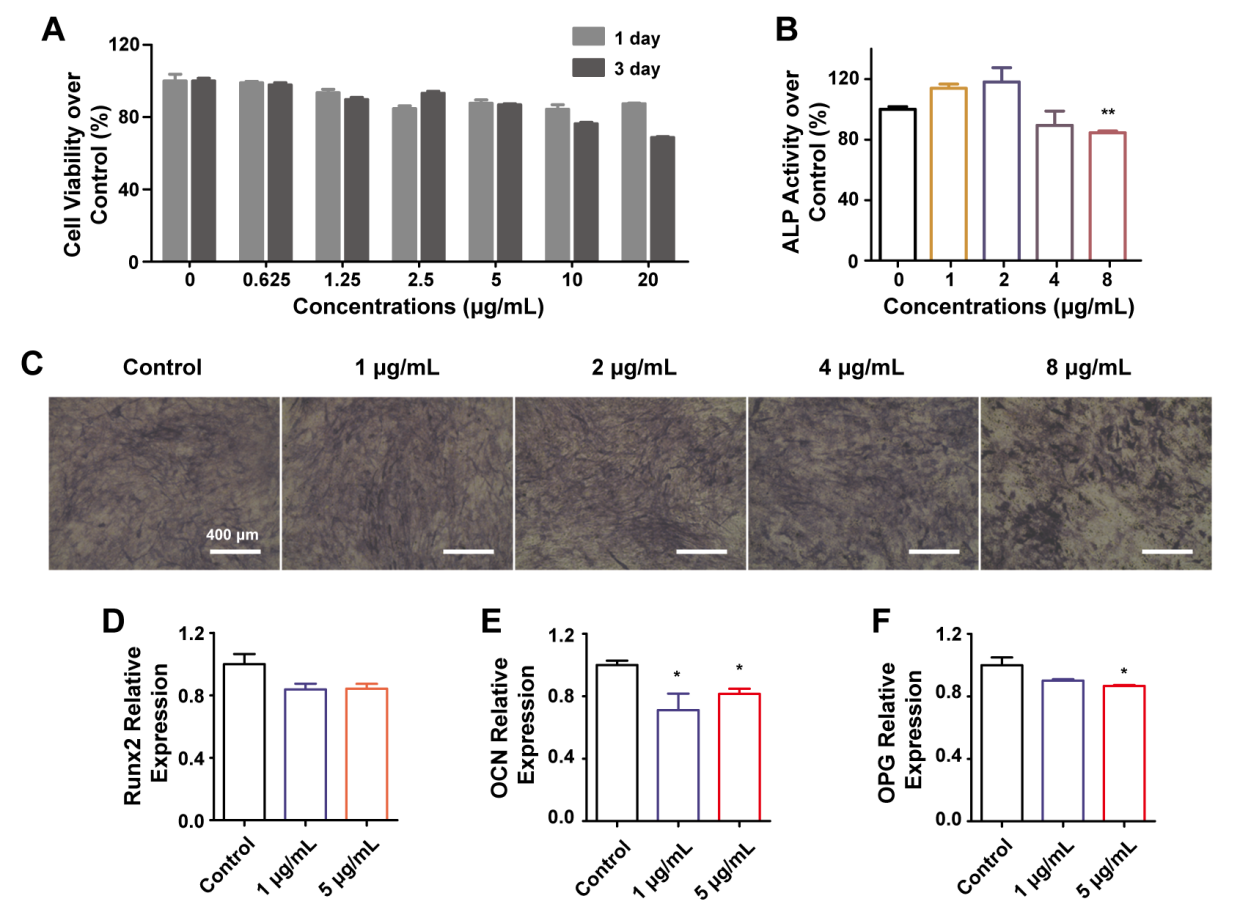


**Figure** **S2.** Effects of MWCNTs on MC3T3-E1 cells. (A) Cell viability after the treatment of different concentrations of MWCNTs for 1 and 3 days. (B) ALP activity. (C) Mineralized nodules of alizarin red staining after being treated with MWCNTs for 21 days. The gene expression of Runx2 (D), OCN (E), and OPG (F). *P<0.05, **P<0.01, and ***P<0.001 represent the comparison with the control group.


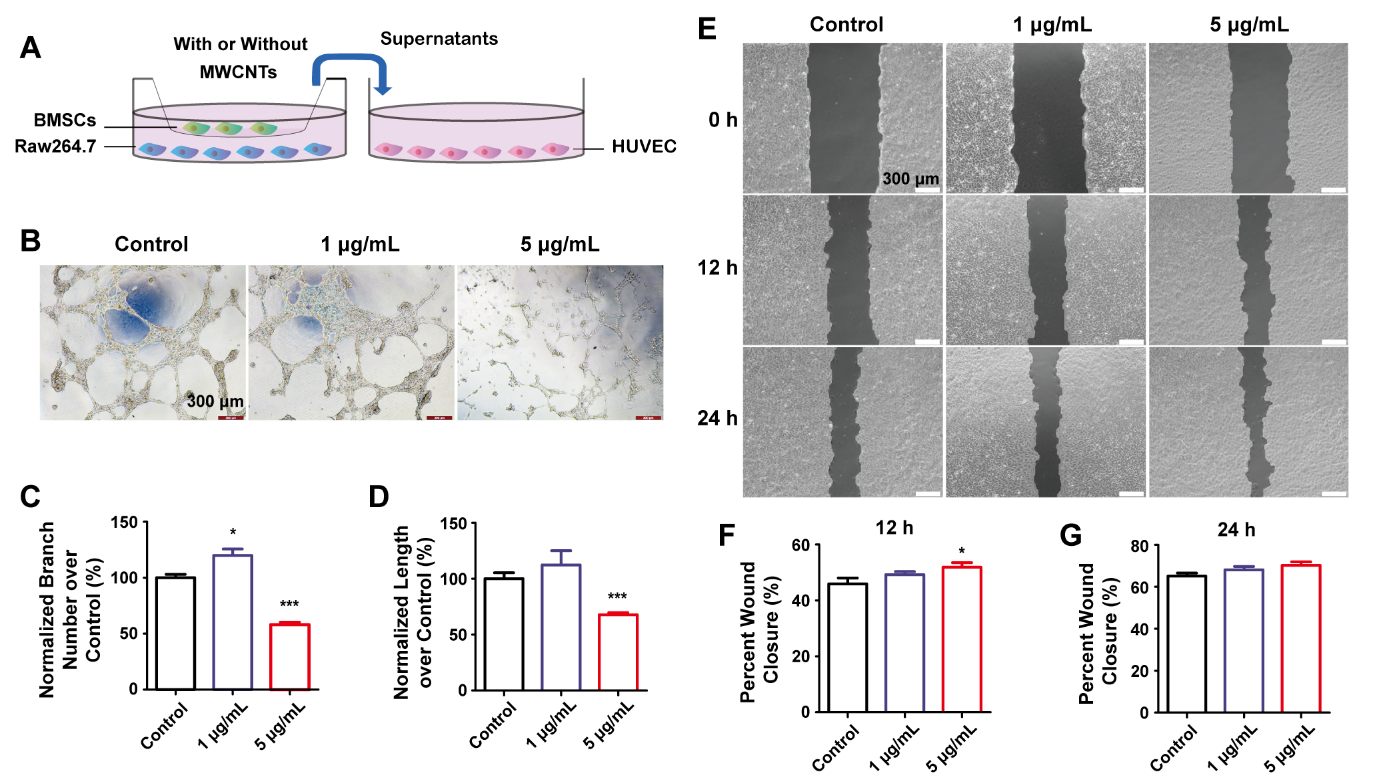


**Figure** **S3.** The angiogenesis of MWCNTs on the co-culture of BMSCs-Raw264.7-HUVECs. (A) The illustration of BMSCs-Raw264.7-HUVECs co-culture system. (B) The capillary-like network of HUVECs was treated with 1 and 5 μg/mL MWCNTs. The number of branches (C) and total length (D) are quantified from (B) by ImageJ software. (E) The migration of HUVECs with the treatment of MWCNTs in the supernatant of BMSCs and Raw264.7 co-culture system. The quantitative results of HUVECs migration at 12 h (F) and 24 h (G). *P<0.05, **P<0.01, and ***P<0.001 represent the comparison with the control group.

**References**

1. Liu D, Yi C, Zhang D, Zhang J, Yang M. Inhibition of proliferation and differentiation of mesenchymal stem cells by carboxylated carbon nanotubes. *ACS Nano* **2010**;4:2185-95.
2. Yan X, Yang W, Shao Z, Yang S, Liu X. Graphene/single-walled carbon nanotube hybrids promoting osteogenic differentiation of mesenchymal stem cells by activating p38 signaling pathway. *Int J Nanomed* **2016**;11:5473-84.
3. Geng H, Chang Y, Bai X, Liu S, Yuan Q, Gu W, Li J, Chen K, Xing G, Xing G. Fullerenol nanoparticles suppress RANKL-induced osteoclastogenesis by inhibiting differentiation and maturation. *Nanoscale* **2017**;9:12516-23.
4. Zhu S, Jing W, Hu X, Huang Z, Cai Q, Ao Y, Yang X. Time-dependent effect of electrical stimulation on osteogenic differentiation of bone mesenchymal stromal cells cultured on conductive nanofibers. *J Biomed Mater Res A* **2017**;105:3369-83.
5. de Paula ACC, Safar GAM, Goes AM, Bemquerer MP, Ribeiro MA, Stumpf HO. Dragging human mesenchymal stem cells with the aid of supramolecular assemblies of single-walled carbon nanotubes, molecular magnets, and peptides in a magnetic field. *BioMed Res Int* **2015**;2015:143504.
6. Li MW, Wei L, Zhou W, He Z, Ran S, Liang J. miR-200a contributes to the migration of BMSCs induced by the secretions of E. faecalis via FOXJ1/NFκB/MMPs axis. *Stem Cell Res Ther* **2020**;11:317-29.
7. Kim HJ, You SJ, Yang DH, Eun J, Park HK, Kim MS, Chun HJ. Injectable hydrogels based on MPEG-PCL-RGD and BMSCs for bone tissue engineering. *Biomater Sci-UK* **2020**;8:4334-45.
8. Fan X, Teng Y, Ye Z, Zhou Y, Tan W. The effect of gap junction-mediated transfer of miR-200b on osteogenesis and angiogenesis in a co-culture of MSCs and HUVECs. *J Cell Sci* **2018**;131:jcs216135.
